# Propensity score matched comparison of SBRT versus IMRT for the treatment of localized prostate cancer

**DOI:** 10.1007/s13566-015-0237-0

**Published:** 2016-01-23

**Authors:** Caspian Oliai, Matthew Bernetich, Luther Brady, Jun Yang, Alexandra Hanlon, John Lamond, Steven Arrigo, Michael Good, Michael Mooreville, Bruce Garber, Rachelle Lanciano

**Affiliations:** Philadelphia Cyberknife Center, 2010 West Chest Pike, Suite 115, Havertown, PA 19083 USA; Department of Radiation Oncology, Drexel University College of Medicine, 230 North Broad Street, Philadelphia, PA 19102 USA; Claire M Fagin Hall, University of Pennsylvania, 418 Curie Boulevard, 479L, Philadelphia, PA 19104-4217 USA; Department of Radiation Oncology, Delaware County Memorial Hospital, 501 North Lansdowne Avenue, Drexel Hill, PA 19026 USA

**Keywords:** Stereotactic body radiation therapy, Intensity-modulated radiation therapy, Hypofractionation, Prostate cancer, High risk

## Abstract

**Objective:**

Stereotactic body radiation therapy (SBRT) is an attractive option for prostate cancer due to its short treatment duration and cost. In this report, we compare the efficacy and toxicity outcomes of prostate cancer patients treated with SBRT to those who received intensity-modulated radiation therapy (IMRT).

**Methods:**

Two hundred sixty-three patients with localized prostate adenocarcinoma were included, ranging from clinically very low- to high-risk groups. We retrospectively compare consecutive patients treated with SBRT with consecutive patients treated with conventionally fractionated IMRT. For most patients, SBRT was delivered to a total dose of 36.25 Gy in five fractions and IMRT to 75.6 Gy in 42 fractions. To minimize selection bias, we perform propensity score analyses.

**Results:**

The treatment groups became similar after propensity matching with absolute standard bias reduced to ≤0.19. For the first analysis, 5-year actuarial survival was 90.8 and 88.1 % in SBRT and IMRT groups, respectively (*p* = 0.7260), while FFBF was 88.7 and 95.5 %, respectively (*p* = 0.1720). For the second analysis (accounting for risk group), actuarial 5-year survival was 96.7 and 87.1 % in the SBRT and IMRT groups, respectively (*p* = 0.3025), while FFBF was 89.7 and 90.3 %, respectively (*p* = 0.6446). Toxicity did not exceed grade 3 in any of the studied patients. The highest recorded genitourinary toxicity at the time of latest follow-up was grade 2.

**Conclusion:**

Our data support the hypothesis that SBRT has non-inferior efficacy and toxicity rates as IMRT. Given the lower cost and convenience for patients, SBRT may be considered as an alternative treatment for localized prostate cancer.

## **Introduction**

Intensity-modulated radiation therapy (IMRT) is one of the standard radiotherapeutic techniques for the definitive treatment of localized prostate cancer. There are several decades of data supporting excellent biochemical control and overall survival for delivery of conventionally fractionated radiation therapy to the prostate and surrounding tissue at risk, while maintaining acceptable toxicity [1]. IMRT was adopted prior to long-term data from phase 3 trials and demonstrated a superior therapeutic ratio [2–4]. The rationale was based on IMRT being a technical innovation on an already proven treatment modality, conventionally fractionated radiation therapy, delivered by three-dimensional conformal radiation therapy (3DCRT) or two-dimensional conventional techniques (2DCT) with sufficient long-term data to support its use as standard of care.

Stereotactic body radiation therapy (SBRT) is a technologically advanced treatment modality that takes advantage of the low alpha-beta ratio of prostate cancer in order to deliver extreme hypofractionated radiation therapy. As a result, SBRT achieves high biological equivalent doses substantially above those attempted in IMRT.

There has been further interest in SBRT for prostate cancer due to its convenience for patients (duration of 1 to 2 weeks, as compared to 8 to 9 weeks of IMRT) and lower cost [5]. SBRT has been considered a different treatment modality than conventionally fractionated therapy and IMRT due to the complex radiobiological differences of extreme hypofractionation compared to standard fractionation. Late responding normal tissue surrounding the malignant tissue may be more susceptible to late toxicity after receiving high doses delivered in fewer fractions. Therefore, one may argue that extreme hypofractionated schemes require many years of follow-up to accurately assess late toxicity.

It is ideal to adopt a new therapeutic modality that is cheaper and more convenient for patients once randomized trials demonstrate non-inferior outcomes and acceptable toxicity. However, by the time phase 3 trial data mature in localized prostate cancer, we may be faced with new scientific and bureaucratic pressures that push prostate cancer treatment in a different direction. Reliance on randomized trials alone is not practical, given the past difficulty in enrolling patients into phase 3 trials that compare prostate cancer treatment modalities head-to-head. The logic used to adopt IMRT as standard of care by means of long-term data from 3DCRT/2DCT can be similarly applied to SBRT, by using data from high-dose rate (HDR) monotherapy as a surrogate, in which data supporting the latter [6–11] justify use of the former.

Therefore, a balance is required which allows new technologies to be offered to patients prior to maturation of long-term data from phase 3 trials. This can be achieved for prostate SBRT through evidenced-based treatment protocols based on long-term data from HDR monotherapy and 5-year data from phase 2 trials of SBRT. Irrespective of the controversy surrounding SBRT, there have been early publications which report promising clinical efficacy of SBRT for localized prostate cancer, along with low rates of toxicity [12]. However, the literature is limited by short-/intermediate-term follow-up. As we await long-term data from ongoing randomized studies, there has been a high level of interest to compare extreme hypofractionation to conventionally fractionated approaches.

We seek to enhance the perspective on this topic by comparing our experience with SBRT and IMRT. Yu et al. published an analysis of Medicare beneficiaries comparing toxicity and cost between SBRT and IMRT.

At 2-year follow-up, they demonstrated a small but statistically significant increase in toxicity in patients who received SBRT as compared to IMRT, while the former had a substantially lower financial cost [5]. Our current study similarly compares SBRT toxicity to IMRT, but additionally analyzes treatment outcome by means of biochemical control and overall survival utilizing propensity score modeling.

## **Methods**

### Stereotactic body radiation therapy

All 142 consecutive men with localized prostate cancer treated between 2007 through 2012 at our satellite radiation oncology center with SBRT as monotherapy were included in this IRB-approved study. The CyberKnife system with multi-plan inverse treatment planning and motion tracking of internal fiducials was used. Treatment planning began with fiducial placement into the prostate. A CT scan was obtained 10–14 days later. T2 fast echo MRI was obtained and three-dimensionally registered by fiducials to the CT in the majority of patients.

The clinical target volume (CTV) was the prostate for low-risk patients and the prostate plus proximal seminal vesicle base for most intermediate- and high-risk patients. Pelvic lymph nodes were never targeted with SBRT. Five fractions were prescribed to the planning target volume (PTV) that consisted of the CTV with a 5-mm margin in all directions except 3 mm posteriorly. The dose delivered changed over the time of the study. Initially, patients received 35 Gy, followed by 37.5 Gy, and at the time of this publication to 36.25 Gy, driven by data available at that time and standard protocol established by Accuray. At least 95 % of the PTV received the prescribed dose normalized to the 75–85 % isodose line (dose heterogeneity 17–33 %). Less than 1 cm^3^ of rectum received 36 Gy, 50 % of the prescribed dose could not cross the posterior rectal wall, and <10 cm^3^ of bladder received 37 Gy. The average CTV and PTV were 56.9 cm^3^ (std dev 27.7 cm^3^) and 98.0 cm^3^ (std dev 48.9 cm^3^), respectively. Orthogonal 120-kV x-ray image pairs were obtained throughout treatment for use in motion tracking. Real-time prostate position was locked on by the relative fiducial position on the x-rays. For those patients with evenly distributed fiducials in the prostate quadrants, the prostate’s rotation was also tracked and corrections were made in real time.

### Intensity-modulated radiation therapy

All 121 consecutive men with localized prostate cancer between 2007 through 2012 at our hospital radiation oncology center treated with IMRT were included in this IRB-approved study. Of note, IMRT and SBRT are delivered at separate geographic sites within the same radiation oncology department/hospital. The CTV was defined as the prostate with 8-mm margin in all directions except 5 mm posteriorly in low-risk patients, prostate plus seminal vesicles with 5- to 8-mm margin for intermediate-risk patients, and prostate plus seminal vesicles plus true pelvic lymph nodes with 5- to 8-mm margin for high-risk patients. Dose constraints to the rectum were defined as V65 <17 % and V40 <35 %, while the bladder constraint was V65 <25 % and V40 <50 %. All patients were simulated in the supine position with immobilization, full bladder, and empty rectum. CT and MRI treatment planning was completed with merging of the images for contouring of the prostate. Image guidance was provided by BAT ultrasound pretreatment daily and patients were treated with full bladder daily. Usually, five to seven isocentric beams were utilized to treat the prostate with 6 MV photons optimized with an inverse optimization algorithm with at least 95 % of the prostate receiving the prescribed dose. The majority of patients received at least 75.6 Gy to the prostate in 1.8 Gy per fraction. Of those that received less than 75.6 Gy, the majority were stratified into either the very-low-risk or low-risk groups.

### Analysis

PSA nadir to date is defined as the lowest PSA value following radiotherapy. Benign PSA bounce was defined as a PSA rise of ≥0.2 ng/mL above its previous nadir with subsequent decline to that nadir or lower. Biochemical failure (BF) was assessed using the nadir +2 (Phoenix) definition. Toxicity was assessed using the Radiation Therapy Oncology Group (RTOG) criteria; acute toxicity occurred within 3 months and late toxicity >3 months following treatment. Risk group was based on the National Cancer Center Network (NCCN) version 2.2014 classification: very low, low, favorable intermediate (one intermediate risk factor), unfavorable intermediate (more than one intermediate risk factor), and high. Since there were few very high risk patients, they were included in the high-risk group.

### Statistics

**Fig. 1:**
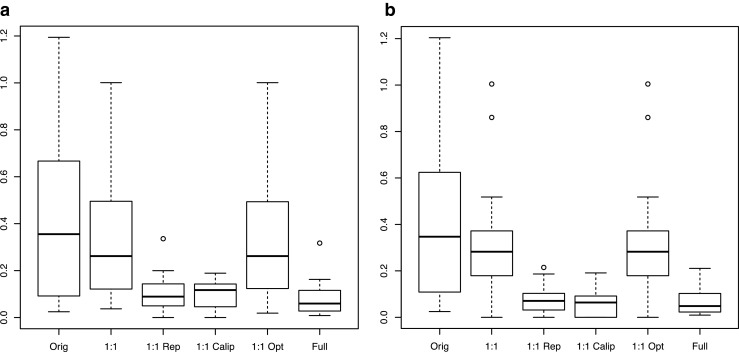
Box plots of different matching approaches: **a** matched on multiple covariates, **b** matched on risk groups

**Fig. 2:**
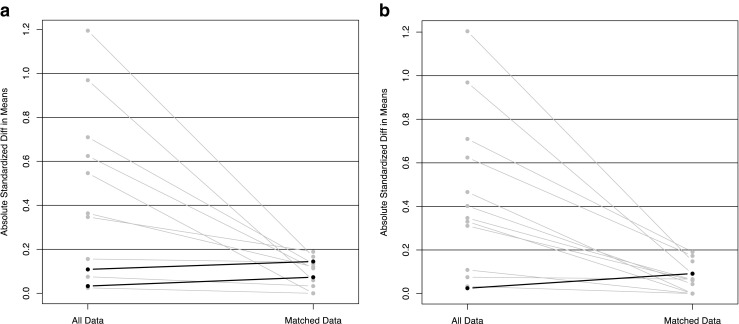
Graphs of unmatched and matched absolute standard bias: **a** Matched on multiple covariates, **b** matched on risk groups

The overall sample is described using measures of central tendency (mean and median) and variation [standard deviation and interquartile range (IQR)], and compared by treatment group using two-sample *t* tests and Fisher exact tests, as appropriate. To minimize selection bias inherent in treatment group allocation, propensity score modeling was used to match the two groups using a logistic regression approach [13]. To evaluate the robustness of the choice of matching covariates, two sets of characteristics were used for the propensity score modeling: (1) individual patient and tumor characteristics including age, tumor stage, Gleason score, pretreatment PSA, treatment year, and androgen deprivation therapy (ADT) use; and (2) NCCN version 2.2014 risk group, age, treatment year, and ADT use. For each set of matching covariates, various propensity score modeling strategies were employed, including simple 1:1, 1:1 with replacement, 1:1 with caliper, 1:1 optimal, full matching, and sub-classification. The methods were compared in terms of bias reduction using box plots (Fig. 1) and overlap of the propensity scores using box plots. An absolute standard bias measure <0.20 is considered small, and sufficient overlap is required for the propensity scores [14]. As seen in Fig. 1, the method demonstrating optimal bias reduction was 1:1 with caliper for both sets of matching covariates, and was thus chosen as the basis for outcome analysis. Figure 2 demonstrates that bias was reduced to <0.20 for all variables used in both of the matching analyses. Kaplan-Meier estimates of overall survival and freedom from biochemical failure were used to describe the patients overall, and comparisons were accomplished using log-rank statistics [15, 16].

## Results

### Patient and treatment characteristics

The outcomes of 263 patients were analyzed, with median follow-up of 43 months (IQR 24–58). The SBRT treatment group of 142 consecutively treated patients had median follow-up of 34 months (IQR 22–54). The IMRT treatment group of 121 consecutively treated patients had median follow-up of 51 months (IQR 33–64). SBRT doses were 35.0 Gy in five fractions (4 %), 36.25 Gy in five fractions (75 %), and 37.5 Gy in five fractions (21 %) of patients. IMRT was delivered in conventional fractionation with 1.8–2.0 Gy per fraction, median dose 75.6 Gy delivered in 42 fractions, specifically, 6 % 79.2 Gy, 18 % 78 Gy, 59 % 75.6 Gy, and 17 % 72 Gy.

The SBRT group comprised patients with younger age and lower risk disease determined by the pooled results of a two-sample *t* test and Fisher’s exact test, respectively, as compared to the IMRT group (Table 1).

**Table 1:** Patient and tumor characteristics for unmatched data

	**SBRT**	**IMRT**	***p*** **value**
**Sample size (** ***n*** **)**	142	121	
**Age (years)**			<0.0001
Mean (std dev)	66.9 (8.0)	71.6 (6.7)	
Median (IQR)	67 (61, 73)	72 (67, 77)	
**Gleason score,** ***n*** **(%)**			<0.0001
5,6	76 (53.5 %)	34 (28.1 %)	
7	54 (38.0 %)	61 (50.4 %)	
8,9,10	12 (8.5 %)	26 (21.5 %)	
**Pre-treatment PSA (ng/mL)**			0.0907
Mean (std dev)	8.1(7.7)	11.0 (18.8)	
Median (IQR)	5.7 (4.4, 8.3)	6.2 (5.0, 9.5)	
**NCCN risk group** ***n*** **(%)**			<0.0001
Very low	28 (19.7 %)	9 (7.4 %)	
Low	33 (23.2 %)	13 (10.7 %)	
Favorable Intermediate	50 (35.2 %)	39 (32.2 %)	
Unfavorable intermediate	13 (9.2 %)	28 (23.1 %)	
High	18 (12.7 %)	32 (26.5 %)	
**ADT use** ***n*** **(%)**	40 (28.2 %)	87 (71.9 %)	<0.0001
**Treatment year** ***n*** **(%)**			0.0007
2007	12 (8.5 %)	28 (23.1 %)	
2008	36 (25.4 %)	32 (26.5 %)	
2009	25 (17.6 %)	25 (20.7 %)	
2010	24 (16.9 %)	19 (15.7 %)	
2011	29 (20.4 %)	7 (5.8 %)	
2012	16 (11.3 %)	10 (8.3 %)	
**Tumor stage** ***n*** **(%)**			0.0001
T1b, T1c, T2a, T2b	132 (93 %)	91 (75.8 %)	
T2c, T3, T3a	10 (7 %)	29 (24.2 %)	

*Age and pretreatment PSA *p* values computed using the pooled *p* value of two-sample *t* tests. Gleason score, NCCN risk group, ADT use, treatment year, and tumor stage *p* values computed using Fisher’s exact test (two-sided *p* value for ADT use and tumor stage)

Patients treated with IMRT were more than twice as likely to receive ADT compared with SBRT, which was consistent for each risk group.

### Unmatched outcome

At most recent follow-up, there were no deaths from prostate cancer in either treatment group. All patients died of causes unrelated to malignancy in the SBRT group, whereas 9 of 15 patients in the IMRT group died of cancer-related causes not associated with prostate adenocarcinoma—primaries of the lung, pancreas, esophagus, and leukemia. Metastatic progression occurred after biochemical failure in three of six patients in the SBRT group and three of nine patients in the IMRT group.

Treatment with both modalities was well tolerated. Acute and late genitourinary (GU) toxicity experienced after treatment did not exceed grade 3. The most severe acute and late gastrointestinal (GI) toxicity was grade 2. All grade 3 toxicities subsided at most recent follow-up. Grade 2 GU toxicity persisted in 12 and 14 % of patients in the IMRT and SBRT groups, respectively. Grade 2 GI toxicity persisted in 1 and 3 % of IMRT and SBRT groups, respectively.

Grade 3 erectile dysfunction (ED) was defined as inability to achieve an erection sufficient for intercourse despite the use of medications or the need for penile prosthesis. The analysis of this particular outcome excluded patients on long-term ADT and those who had grade 3 ED at baseline. At most recent follow-up, grade 3 ED persisted in 17 % of those who received IMRT and in 6 % of those who received SBRT.

### Matched analyses

**Fig. 3:**
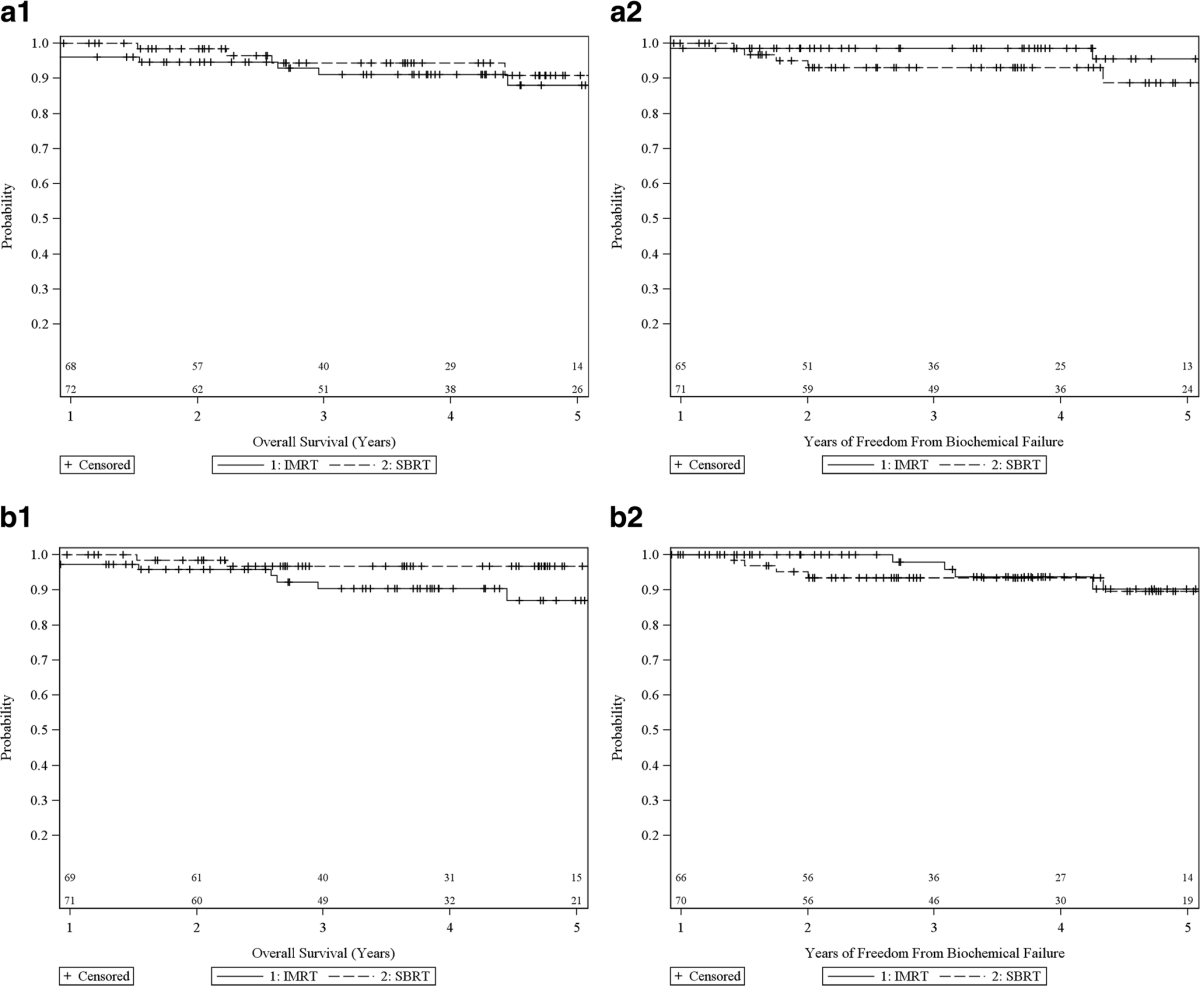
Five-year actuarial survival and freedom from biochemical failure: **a1**, **a2** matched on multiple covariates; **b1**, **b2** matched on risk groups

In order to minimize selection bias resulting from confounding patient and treatment differences between the SBRT and IMRT treatment groups, we performed two additional propensity score analyses which matched patients between the two groups. The first analysis relied on 1:1 propensity score matching with a caliper using age, Gleason score, pretreatment PSA, treatment year, tumor stage, and ADT use. The resulting treatment groups became similar, with the absolute standard bias reduced to ≤0.19 for all covariates (Table 2). For this sample, the 5-year actuarial survival (Table 3 and Fig. 3) was 90.8 and 88.1 % in the SBRT and IMRT treatment groups, respectively (*p* = 0.7260, overall comparison between curves). For the same sample, the 5-year actuarial FFBF was 88.7 and 95.5 % in the SBRT and IMRT treatment groups, respectively (*p* = 0.1720 overall, Table 3 and Fig. 3).

**Table 2:** Matched and unmatched means and absolute standard biases based on covariates

	**Unmatched mean**		**Absolute standard bias**	**1:1 Matched with caliper mean, 1st group (all covariates)**		**Matched absolute standard bias**	**1:1 Matched with caliper mean, 2nd group (on risk group)**		**Matched absolute standard bias**
	**IMRT**	**SBRT**		**IMRT**	**SBRT**		**IMRT**	**SBRT**	
**Age**	71.6281	66.9085	0.7096	70.3067	69.4133	0.1343	70.8108	69.5405	0.191
**Gleason score**			0.5463			0			
**5, 6**	0.2810	0.5352		0.3867	0.36				
**7**	0.5041	0.3803		0.44	0.4933				
**8, 9, 10**	0.2149	0.0845		0.1733	0.1467				
**Pretreatment PSA**	10.9962	8.0702	0.1559	10.9892	8.3375	0.1413			
**EOT year**									
**2007**	0.2314	0.0845	0.3469	0.2267	0.1467	0.1889	0.1351	0.1622	0.0683
**2008**	0.2645	0.2535	0.0247	0.2667	0.2667	0	0.3108	0.2703	0.0915
**2009**	0.2066	0.1761	0.0752	0.2	0.1867	0.0328	0.2027	0.1757	0.0665
**2010**	0.157	0.169	0.0328	0.1333	0.16	0.073	0.2027	0.2027	0
**2011**	0.0579	0.2042	0.6244	0.0933	0.12	0.1137	0.0811	0.1216	0.1729
**2012**	0.0826	0.1127	0.1086	0.08	0.12	0.1447	0.0676	0.0676	0
**Tumor stage**			0.3636			0.1205			
T1b, T1c, T2a, T2b	0.7583	0.9296		0.80	0.88				
T2c, T3, T3a	0.2417	0.0704		0.1867	0.12				
**ADT use**	0.719	0.2817	0.9689	0.56	0.5333	0.0591	0.5676	0.527	0.0898
**5 risk groups**									
**High**	0.2645	0.1268	0.3109				0.2162	0.1892	0.061
**Unfavorable intermediate**	0.2314	0.0915	0.3303				0.1351	0.1351	0
**Favorable intermediate**	*	*	*				*	*	*
**Low**	0.1074	0.2324	0.4018				0.1622	0.1757	0.0435
**Very low**	0.0744	0.1972	0.4661				0.1081	0.1081	0

*Reference group

**Table 3:** Unmatched and 1:1 caliper matching with 5-year actuarial survival

			**1**	**2**	**3**	**4**	**5**	**Log-rank** ***p*** **value**
**Overall survival**	All patients unmatched	All patients (*N* = 263)	98.8 %	97.5 %	94.2 %	92.5 %	89.6 %	
	Matched using all covariates	SBRT (*N* = 75)	100 %	98.4 %	94.4 %	94.4 %	90.8 %	0.7260
		IMRT (*N* = 75)	96.0 %	94.6 %	91.1 %	91.1 %	88.1 %	
	Matched using risk group	SBRT (*N* = 74)	100.0 %	98.5 %	96.7 %	96.7 %	96.7 %	0.3025
		IMRT (*N* = 74)	97.3 %	95.8 %	90.4 %	90.4 %	87.1 %	
**Freedom from biochemical failure**	All patients unmatched	All patients (*N* = 263)	99.6 %	97.3 %	96.1 %	93.8 %	91.7 %	
	Matched using all covariates	SBRT (*N* = 75)	100 %	93.1 %	93.1 %	93.1 %	88.7 %	0.1720
		IMRT (*N* = 75)	98.6 %	98.6 %	98.6 %	98.6 %	95.5 %	
	Matched using risk group	SBRT (*N* = 74)	100.0 %	93.4 %	93.4 %	93.4 %	89.7 %	0.6446
		IMRT (*N* = 74)	100.0 %	100.0 %	98.0 %	93.7 %	90.3 %	

*Matched using all covariates: SBRT and IMRT treatment groups matched by treatment year, t-stage, age, GS, pretreatment PSA, ADT use

*Matched using risk group: SBRT and IMRT treatment groups matched by NCCN v2.2014 risk group, ADT use, age, treatment year

Similarly, we generated a propensity score analysis using 1:1 matching with a caliper to account for differences in NCCN version 2.2014 risk group, ADT use, age, and treatment year. As a result of the matching, the resulting treatment groups again became similar, for which the absolute standard bias was reduced to ≤0.19 for all risk groups (Table 2). For this sample, the 5-year actuarial of overall survival (Table 3 and Fig. 3) was 96.7 and 87.1 % in the SBRT and IMRT treatment groups, respectively (*p* = 0.3025, overall comparison between curves). For the same sample, the 5-year actuarial of FFBF was 89.7 and 90.3 % in the SBRT and IMRT treatment groups, respectively (*p* = 0.6446 overall, Table 3 and Fig. 3).

## Discussion

Given the retrospective observational nature of our study, two separate matching methods were used to reduce bias introduced by confounding prognostic factors with treatment, and thus balance differences between treatment groups. Results from 1:1 propensity score matching with caliper based on age, Gleason score, pretreatment PSA, treatment year, tumor stage, and ADT use demonstrate no statistically significant differences in outcomes between SBRT and IMRT treatment groups. Consistent with these findings, similar matching based on NCCN risk group, ADT use, age, and treatment year also demonstrate no statistically significant differences in outcomes between treatment groups. Ideally, propensity score matching seeks to minimize differences between imbalanced groups in order to create samples similar to those in a prospective randomized study. Thus, separate analyses produced similar results, in which there was no difference in overall survival or FFBF in our patient population.

A common critique of SBRT for prostate cancer is that adopting it as routine practice should await long-term results from randomized phase 3 trials. It has been suggested that an ongoing Swedish phase 3 trial of hypofractionated radiotherapy delivered in >5 fractions (ISRCTN45905321) [17] will help shed light on this topic once published [18]. Several phase 3 trials of hypofractionation in >5 fractions have been reported, albeit using moderate hypofractionation, of which four of five show no significant difference in rates of late toxicity [19–23]. The trial with the longest follow-up (90 months) demonstrated that a conventionally fractionated scheme was an independent prognostic factor for worse late GU toxicity [21].

Several prospective studies of SBRT have reported high rates of biochemical control with acceptable toxicity. King et al. demonstrate the favorable therapeutic ratio in a consortium of patients from phase 2 trials who were treated between 2003 and 2011 at eight institutions. Five-year bRFS was achieved in 95, 84, and 81 % of low-, intermediate-, and high-risk patients, respectively. The use of ADT and SBRT dose did not significantly affect bRFS even after stratifying by risk group [12]. In a separate publication, King et al. describe patient-reported quality of life (QOL) from a sample of 864 patients from four phase 2 studies that evaluate SBRT. They demonstrated urinary and bowel QOL decline within the first 3 months after treatment, recovering by 6 months, remaining stable at 3 years’ follow-up, and subsequently achieving superior QOL as compared to baseline [24]. This trend was independent of severity of acute toxicity and duration of ADT use. Katz et al. compared similar prospective QOL measures in patients who received SBRT and radical prostatectomy. Both modalities resulted in decline of urinary and bowel QOL at 1 month, with an upward trend at later time points. After SBRT, QOL recovered by 6 months to levels not significantly different than baseline (latest 36 months), whereas QOL after surgery remained significantly lower compared to baseline at all time points [25]. Katz et al. series of 477 patients with low- and intermediate-risk prostate cancer treated with SBRT demonstrated a 7-year actuarial FFBF of 95.6 and 89.6 % for low- and intermediate-risk patients, respectively. No late grade 3 GI toxicity was noted and late GU toxicity was limited to 1.7 % [26].

Comparison of SBRT to IMRT for localized prostate cancer has been published in one other study to date [5]. An analysis of Medicare beneficiaries in the United States (age >65) treated from 2008 through 2011 showed greater toxicity associated with SBRT than with IMRT. The absolute difference in toxicity at the latest time point of 24 months was 8 % (*p* = 0.01). However, toxicity was neither graded nor reported subjectively. The radiation treatment platform used for SBRT was not reported nor was there a dose/volume analysis presented. It was reported based on claims submitted via international coding of disease (ICD-9). Differences in toxicity may have little clinical relevance if, for example, an 8 % difference comprised mostly of grade 1 or 2 toxicity. Furthermore, late toxicity will be inherently missed in a study which does not exceed 24 months’ follow-up. The Medicare analysis does not report PSA outcome or survival, as this information is not obtainable through the Medicare database. Nonetheless, Yu et al. provide an important perspective on two distinct treatment modalities. The financial cost was reported to be substantially lower for SBRT than IMRT, with mean cost for a full course of IMRT $7348 more than SBRT.

Our current series complements the Medicare analysis by Yu et al. Together, our publications enable a hypothesis to be generated for future prospective trials, in which SBRT may be non-inferior to IMRT in regard to GU/GI toxicity, survival, and biochemical outcome, while having a substantially lower financial cost. Limitations of our study include the selection bias that arises in retrospective observational series. However, we attempt to minimize such bias by incorporating two statistical propensity-matched analyses.
